# Mother–Child Relationships in U.S. Latinx Families in Middle Childhood: Opportunities and Challenges in the 21st Century

**DOI:** 10.3390/socsci11010008

**Published:** 2021-12-24

**Authors:** Kimberly A. Updegraff, Adriana J. Umaña-Taylor, Daye Son, Karina M. Cahill

**Affiliations:** 1 School of Social and Family Dynamics, Arizona State University, Tempe, AZ 85287, USA;; 2 Harvard Graduate School of Education, Harvard University, Cambridge, MA 02138, USA;

**Keywords:** culture, Hispanic, Latino/a/x, middle childhood, mothers/mother–child relations

## Abstract

The 21st century has brought unique opportunities and challenges for parents, and this is particularly true for Latinx families, whose children comprise more than one-fourth of the school-age population in the U.S. today. Taking an ecological and strengths-based approach, the current study examined the role of mothers’ cultural assets (familism values, family cohesion) and challenges (economic hardship, ethnic–race-based discrimination) on children’s educational adjustment in middle childhood, as well as the indirect role of mother–child warmth and conflict in these associations. The sample included 173 Latinx mothers and their middle childhood offspring (i.e., 5th graders and younger sisters/brothers in the 1st through 4th grade). Mothers participated in home visits and phone interviews and teachers provided ratings of children’s educational adjustment (academic and socioemotional competence, aggressive/oppositional behaviors). Findings revealed family cohesion was indirectly linked to children’s educational adjustment via mother–child warmth and conflict, particularly for younger siblings. Discussion focuses on the culturally based strengths of Latinx families and highlights potential implications for family-based prevention in middle childhood.

## 1. Introduction

The 21st century has been characterized by significant growth in ethnic–racial minoritized groups in the U.S. population (Krogstad 2019), with Latinxs representing the largest ethnic–racial minoritized group (Krogstad and Noe-Bustamante 2021) and accounting for more than half of the population growth since the turn of the century (Flores 2017). Further, the significant growth of ethnic–racial minoritized groups alongside more modest growth in the White population means that the U.S. is projected to transition to a ‘majority-minority’ nation during the 21st century, with half or more of the population identifying as members of ethnic–racial minoritized groups (Colby and Ortman 2015). This transition has already occurred among U.S. children: 50% are from ethnic–racial minoritized backgrounds (Child Trends 2018). In the K-12 school system, 27% of students are Latinx (National Center for Education Statistics 2021), and Latinxs are expected to be one-third of the U.S. workforce by 2060 (Toossi 2016). Thus, research that examines the experiences of Latinx parents, including both the opportunities and challenges they face in raising their children, is critical in shaping prevention programs that are relevant to this substantial segment of U.S. families.

A universally important task for U.S. parents today is supporting their children’s educational adjustment (Van Hook and Glick 2020), broadly defined to include academic, socioemotional, and behavioral functioning in the school setting (Magnuson et al. 2016). The benefits of children’s successful adjustment in educational settings are well-documented and far reaching: children with higher academic achievement and better socioemotional and behavioral functioning in middle childhood are more likely to complete high school (Magnuson et al. 2016; Rabiner et al. 2016), which has positive implications for postsecondary education and future employment and earnings (National Center for Education Statistics 2021), as well as for physical and mental health (Lee et al. 2016; Krueger et al. 2019). In Latinx families, parents’ values and aspirations can play a significant role in their children’s educational success (Fuligni and Fuligni 2007; Stepler 2016). In particular, strong family-oriented values and behaviors in Latinx culture have been identified as potential cultural assets that may engender family supports for children’s education and underlie children’s effort and motivation to succeed in school (Cahill et al.; Stein et al. 2014).

In addition to culturally informed strengths, however, Latinx parents can also face challenges, some of which are unique to their sociocultural backgrounds and circumstances (Leidy et al. 2010). For instance, the seminal work by Lareau (2011) notes that supporting children educationally requires navigating institutions designed for middle class (primarily White) families, and that this poses greater challenges for families with fewer resources and from ethnic–racial minoritized groups. Latinx children are disproportionately likely to attend economically disadvantaged schools (Hussar et al. 2020) and to live in poverty (Shrider et al. 2021). Among parents who have immigrated to the U.S., additional stressors come from the cultural adaptation process, language barriers, and limited experience with the U.S. educational system (Suarez-Orozco et al. 2010); challenges also emerge from being the target of racism and discrimination (Ayón and Garcia 2019; García Coll and Szalacha 2004). A particularly notable challenge of the 21st century has been anti-immigrant sentiments and policies targeting Latinx populations (Leidy et al. 2010; Roche et al. 2018; Torres et al. 2018). These challenges and stressors may contribute to educational disparities among U.S. Latinx individuals, including higher rates of school dropout and lower rates of college enrollment and bachelor’s degree attainment, relative to Black, White, and Asian American individuals (American Psychological Association 2012; Krogstad 2016).

A long-standing deficit-oriented approach to the study of ethnic-racial minoritized youth has impeded the field’s understanding of Latinx families (Perez-Brena et al. 2018), although the introduction of the integrative model of minority youth development (García Coll et al. 1996) brought culture to the forefront of developmental science and called for scholars to move beyond a focus on youth maladjustment and to recognize culturally based strengths. This perspective led to important changes and notably more strengths-based framing of research on ethnic–racial minoritized youth. In an important parallel, however, the literature on parenting among ethnic–racial minoritized families has been characterized by a similar deficit-oriented perspective, with research focused almost exclusively on parenting during adolescence, a developmental period noted for increases in problem behaviors (Rescorla et al. 2016; Wheeler et al. 2017) and declines in school engagement (Benner and Graham 2009). In contrast, little research has examined the role of Latinx parenting in *middle childhood* (for a notable exception, see Domenech Rodriguez et al. 2009).

Middle childhood is a time when children begin formal schooling, have increased opportunities for social interactions outside the home, make substantial gains in educational skills, learn to comply with teachers’ expectations, and form relationships with peers (Collins and Madsen 2019; Votruba-Drzal 2006). Parents’ role in supporting their children’s educational adjustment in middle childhood has the potential to provide a critical foundation for future success, but the specific cultural and parenting mechanisms that are linked to Latinx children’s educational adjustment in middle childhood are not well-understood. To address these gaps, this study drew on culturally informed perspectives (García Coll et al. 1996; Calzada et al. 2012; Stein et al. 2014) to examine the role of Latinx mothers’ cultural assets (i.e., familism values, family cohesion) and challenges (i.e., ethnic-racial discrimination, economic hardship) in their children’s educational adjustment via mother–child relationship qualities (i.e., warmth, conflict) in middle childhood. This study has the potential to broaden the field’s understanding of how parenting is embedded within the cultural and ecological context of the 21st century, and may inform prevention programming *prior* to adolescence and the onset of significant adjustment difficulties for some youth.

### 1.1. Culturally Informed Assets and Latinx Children’s Educational Adjustment

Beginning with cultural assets, we considered mothers’ *familism values*, defined as their endorsement of values regarding family as a source of support, obligation, and referent (Knight et al. 2010), and *family cohesion* (i.e., family as a source of shared activities, interests, and connections; Olson et al. 1982). A central feature of culturally informed models of parenting is the role of parents’ cultural values in shaping their parenting goals and practices and defining desired outcomes for their children (Calzada et al. 2012; Harwood et al. 2002). More specifically, Stein et al.’s (2014) cultural developmental model outlines how familistic attitudes and behaviors manifest in parenting/parent–youth relationships and children’s adjustment from early childhood through adolescence. In middle childhood, familism is hypothesized to be reflected in positive parent–youth relationships, such as warm and responsive parenting and low levels of conflict (Stein et al. 2014), as parents’ strong familism values may promote relationship behaviors that are consistent with their cultural ideal of close and harmonious family relationships (Campos et al. 2008). Supporting these associations, a recent meta-analysis revealed significant associations between familism values and higher levels of family support/warmth and lower levels of family conflict/negativity among Latinx individuals (Cahill et al.).

Stein et al. (2014) also posits that familism in middle childhood manifests in caregivers’ socialization messages emphasizing that children’s behavioral compliance and academic success reflects positively on the family. Thus, as children receive and internalize messages emphasizing familism, they may be motivated to do better in school. Indeed, meta-analytic findings provide support for positive associations between familism values and youth’s more positive educational and behavioral outcomes, with the majority of this work focusing on adolescent and young adult samples (Cahill et al.). Developmentally, children ages 7–11 are coming to understand the importance of their behavior as reflecting on the family, including in contexts outside the home (e.g., with teachers and peers), and beginning the process of internalizing these values (Stein et al. 2014). Supporting these theoretical notions, Morcillo et al. (2011) found that parental familism values were associated with lower rates of antisocial behavior among Puerto Rican children (ages 5 to 9), and family processes partially mediated these associations, particularly in middle childhood (relative to adolescence). These authors proposed that parents’ familism may be especially critical in middle childhood because children have not fully internalized their values (Morcillo et al. 2011). Although the behavioral aspects of familism have received less empirical attention, a study of recent Latinx immigrants (Leidy et al. 2010) revealed that parent-reported family cohesion was associated with more positive socioemotional competencies among children in late childhood/early adolescence (i.e., 9–12-year-olds). Despite the theoretical significance of familism in middle childhood, existing reviews underscore a dearth of studies with this developmental age group (Cahill et al.; Stein et al. 2014). Guided by Stein et al.’s (2014) model, we test the direct associations of mothers’ familism values and family cohesion (as a proxy for familistic behaviors) with children’s educational adjustment, as well as potential indirect associations via mother–child warmth and conflict.

### 1.2. Contextually Informed Risk Factors and Latinx Children’s Educational Adjustment

The integrative model (García Coll et al. 1996) also draws attention to contextual characteristics that may be sources of risk, including social position factors (e.g., socioeconomic status) and mechanisms of oppression (e.g., segregation, discrimination). In this study, we considered mothers’ perceptions of economic hardship and discrimination as factors that may undermine children’s educational adjustment in middle childhood. The family stress model posits that economic hardship impacts youth adjustment via negative effects on parent and family functioning (Conger et al. 1994), and empirical support extends to Latinx families in early childhood (e.g., Derlan et al. 2019) and adolescence (e.g., Delgado et al. 2013; White et al. 2015). Although originally focused on economic strain, the family stress model has been expanded to include parents’ culturally based stressors (Conger et al. 2012). In a study of recent immigrant families of predominantly Cuban and Mexican descent, parents’ culturally based stressors predicted youth’s depression, aggression, and self-esteem via youth’s reports of family functioning in late adolescence (Lorenzo-Blanco et al. 2012). We extend this research to examine mothers’ experiences of economic hardship and ethnic–racial based discrimination as predictors of children’s educational adjustment and indirect associations via mother–youth warmth and conflict in middle childhood.

### 1.3. The Role of Mother–Child Relationship Quality

Warmth and conflict are universal dimensions of parent–youth relationships across a broad range of ethnic, racial, and cultural contexts (Li and Warner 2015; Steinberg 2001), with higher levels of warmth and lower levels of conflict generally being associated with more positive adaptation and fewer adjustment problems (Khaleque 2013; Weymouth et al. 2016). In Latinx culture, where there is a strong emphasis on family-oriented values and practices, parent–youth relationships characterized by high warmth and low conflict may be particularly important for children’s adjustment given their alignment with cultural ideals for close and harmonious family relationships (Campos et al. 2008). Research on the role of parent–youth conflict in Latinx families, although largely based on adolescent samples, suggests particularly negative implications for youth adjustment (Li and Warner 2015; Kuhlberg et al. 2010; Pasch et al. 2006). Examining four Latinx subgroups (Mexican, Cuban, Nicaraguan, Colombian), Li and Warner (2015) found that higher parent–youth conflict was consistently associated with lower ratings of self-esteem among children of immigrants. In another study of Mexican American adolescents, conflict with mothers and fathers was associated with a wide range of internalizing (depression, anxiety) and externalizing (anger, substance use, school misconduct) outcomes (e.g., Pasch et al. 2006). Extending this work to middle childhood, we tested whether mother–child warmth was associated with higher socioemotional and academic competence and lower aggressive/oppositional behavior (Santesteban-Echarri et al. 2017; Taylor et al. 2015); we also tested whether conflict was linked to less optimal educational adjustment.

### 1.4. Moderating Role of Child Gender and Birth Order

Latinx families are characterized by a strong emphasis on gender-differentiated roles and socialization (Umaña-Taylor and Updegraff 2013), which may mean that associations among parents’ cultural assets/challenges, parent–child relationship qualities, and children’s adjustment vary by child gender. Research on youth gender as a moderator of the links among cultural values and practices, parenting, and youth adjustment provide some evidence of stronger associations for girls relative to boys, based on the notion that females are socialized with a stronger emphasis on family roles and relationships in this cultural context (Lorenzo-Blanco et al. 2012; Morcillo et al. 2011; Updegraff et al. 2005). However, this work largely focuses on families with adolescents, and little is known about potential gender differences in middle childhood among Latinx immigrant families (Schroeder and Bámaca-Colbert 2019). Thus, we explored the possibility of gender moderation in middle childhood, expecting that if differences emerged, the associations may be stronger for parents of female versus male children.

A unique strength of our study was the focus on *two* children in each family, as research has documented that children in the same family may experience relationships with their parents differently (e.g., Daniels and Plomin 1985; McHale et al. 2003). Birth order differences in parenting, and their implications for youth adjustment, may emerge for different reasons, including because children are in different developmental periods or because what parents learn from one child has implications for their parenting of the next child (Whiteman et al. 2003). Spillover in family dynamics are another possibility, such as when the older child’s transition to adolescence is linked to increases in parent–child conflict for them and their younger siblings (Shanahan et al. 2007a). There has been attention to within-family differences in parenting, including warmth and conflict, in middle childhood in European American families (Shanahan et al. 2007a, 2007b). Fewer studies have examined parenting of multiple children in Latinx families, with notable exceptions focused on families with adolescents (McHale et al. 2005; Padilla et al. 2016). In this study, we explore whether birth order moderates the direct and indirect associations among parents’ cultural assets and challenges, parent–child relationship quality, and children’s educational adjustment.

### 1.5. The Current Study

The overarching goal of this study was to examine the links among Latinx parents’ culturally informed assets (familism values, family cohesion) and challenges (economic hardship, experiences of discrimination), universal characteristics of parent–child relationships (warmth, conflict), and children’s educational adjustment in middle childhood. Using concurrent reports of parents’ cultural assets, challenges, and parent–child relationship qualities, as well as teachers’ ratings of children’s academic and socioemotional competencies and aggressive/oppositional behaviors, we tested direct associations from parents’ cultural assets and challenges to children’s educational adjustment; we also tested indirect associations via mother–child warmth and conflict. We expected these associations may be stronger for mothers with female, relative to male, children based on gender-differentiated roles in Latinx families (Schroeder and Bámaca-Colbert 2019; Updegraff et al. 2014). We also explored moderation by birth order (i.e., within-family differences). Finally, we controlled for the child’s age to account for the age span of children across middle childhood (i.e., 6 to 11 years of age), and for mothers’ immigrant status, given anti-immigrant sentiments and policies, particularly in the local context of the present study (Santos et al. 2018; Toomey et al. 2014).

## 2. Method

### 2.1. Participants

Participants were 173 Latinx mothers who participated in the baseline phase of a larger clinical trial (NCT 03706014) testing the efficacy of a family-based, sibling-focused prevention program. Eligibility for participation in the clinical trial included having a 5th grader and younger sibling (1st–4th grade) of Latinx origin who lived together for at least three years, attended the same elementary school, and did not have significant disabilities that would prevent participation; children also needed a primary caregiver who spoke English or Spanish and was willing to participate. For 97% of children, the primary caregiver was their biological mother.

To recruit families, letters and recruitment flyers in Spanish and English were distributed to parents/caregivers of Latinx 5th graders who had a sibling in 1st through 4th grade in the same school. All ten participating schools had a Title I designation, and the percentage of students eligible for free/reduced lunch ranged from 78% to 93% across schools. A total of 396 letters were sent, and follow-up calls to assess eligibility and interest in participation were conducted by bilingual staff with 356 families (40 families could not be reached). Of the 356 families, 259 were eligible (73%), 49 refused the screening to determine eligibility (14%), and 48 were ineligible (13%). Of the 259 eligible families, 215 families (83%) provided baseline data, including 185 mothers. Given the goals of the present study, the analytic sample was limited to Latinx mothers (*n* = 173; 93.5% of mothers), and 12 non-Latinx mothers were excluded. In one family, two sibling pairs participated; thus, background characteristics are reported for 173 mothers and their 348 participating children (i.e., 174 fifth graders and 174 younger siblings).

These mothers averaged 36.5 years of age (*SD* = 6.65) and were predominantly of Mexican (90.2%), Guatemalan (3.5%), or Salvadoran origin (2.3%), with the remaining mothers being of another Latinx subgroup (<1%). In terms of racial identification, mothers indicated they were White (37.7%), American Indian/Alaskan Native (2.4%), multiracial (2.4%), or other (57.5%). Most mothers who selected “other” for race specified a panethnic (i.e., Latino/a, Hispanic) or ethnic (e.g., Mexican) label. A majority of mothers (74.6%) were born outside of the U.S and preferred Spanish for their interview language (71.7%). In terms of education level, slightly over half of the mothers did not have a high school degree (55.5% of mothers), and a smaller percentage had a high school degree or GED (31.8% of mothers), with the remainder having some post-high-school education or a college degree. Among participating mothers, 74.6% were married or living with a partner. For those who reported income (89%), median household income was $31,127 (SD = $19,308; range = $1500–$133,000) for an average of 6.1 household members (*SD* = 1.82; range = 3 to 16). A high percentage of families (97.7%) accessed at least one form of public assistance, with the most common being free/reduced price meals at school (92.4%), health insurance (82.1%), and nutritional assistance programs (49.7%). Fifth graders averaged 10.63 years of age (*SD* = 0.41) and younger siblings averaged 8.24 years of age (*SD* = 1.06). Children were identified by mothers as female (50.3%) or male (49.7%).

### 2.2. Procedure

Data were collected via home visits and phone interviews with mothers, and self-administered surveys were completed by teachers. First, each family was visited at home by two project staff (at least one bilingual) to describe the project and complete informed consent/assent and a family background questionnaire. All materials were read aloud to mothers and their answers were recorded on paper surveys. These visits averaged one hour, and families received a $20 honorarium. Following the home visit (2 to 4 weeks), mothers participated in a phone interview in their preferred language lasting an average of 60 min. All questions were read to mothers and their responses were recorded on paper surveys. Each parent received a $35 honorarium for participating in the phone interview. Mothers provided consent for teachers to complete ratings about each participating child. Teachers received $10 for each student packet they completed. All procedures were approved by the Human Subjects Review Boards at Arizona State University and The Pennsylvania State University [Title: Innovative Family Prevention with Latinos; protocol #00006528 and STUDY00010473, respectively] and the Research and Evaluation Board of the participating school district.

### 2.3. Measures

All items were forward translated to Spanish by one person, back translated to English by a second person, and reviewed by a third person. Discrepancies were discussed with the team and resolved following a process of decentering, in which the goal was meaningful, rather than literal, translation (Knight et al. 2009).

#### 2.3.1. Familism Values

Mothers completed the 16-item familism subscale of the Mexican American Cultural Values Scale (Knight et al. 2010). The measure assesses three aspects of familism (i.e., support/closeness, obligations, and family as referent), and items are averaged for an overall mean score. Each item is rated on a 4-point scale ranging from 1 (*Strongly Disagree*) to 4 (*Strongly agree*), with a sample item being “Children should always do things to make their parents happy.” Cronbach’s alpha was 0.84.

#### 2.3.2. Family Cohesion

Mothers completed the cohesion subscale (16 items) of the Family Adaptability and Cohesion Evaluation Scales II (FACES II; Olson et al. 1982); psychometric properties have been established in prior work with Latinx samples (Roosa et al. 1996). The subscale measures family connectedness, with sample items including “Family members like to spend their free time with each other” and “Family members share interests and hobbies with each other.” Each item is rated on a 5-point scale ranging from 1 (*Almost never or never*) to 5 (*Almost always or always*) and items are summed for a scale score. Cronbach’s alpha was 0.79.

#### 2.3.3. Ethnic–Racial Discrimination

Mothers’ experiences of discrimination were assessed with the Everyday Discrimination Scale (EDS), originally developed by Williams et al. (1997) for African American women, using the 9-item adapted version based on Reeve et al. (2011) and Williams et al. (2008). The dimensionality and construct validity of EDS has been empirically supported with the Latinx subsample of the National Latino and Asian Study (Molina et al. 2013). A sample item is “You are treated with less respect than other people are because you are Hispanic/Latino.” Each item is rated on a 5-point scale ranging from 1 (*Almost every day*) to 5 (*Never*). Items were reverse coded so that higher scores indicated more frequent experiences of discrimination (α = 0.92).

#### 2.3.4. Perceived Economic Hardship

Mothers rated their perceptions of economic hardship using a 6-item scale (Howe et al. 1995; Kessler et al. 1988). Mothers were asked how often they experienced each item in the past three months, including having to “Miss payments on your bills,” “Not go to see the doctor or dentist because of cost,” and “Reduce spending on household expenses such as food and clothing.” Each item was rated on a scale of 1 (*Not at all*) to 4 (*A lot*), and items were averaged for a total score (α = 0.74).

#### 2.3.5. Mother–Child Relationship Quality

Mothers completed the parent version of the warmth subscale of the Children’s Report of Parental Behavior Inventory (CRPBI; Schwarz et al. 1985), which includes eight items assessing mothers’ warm and supportive behaviors toward their child (e.g., “I understand [child’s name]’s problems and worries.”). The cross-ethnic and language equivalence (English to Spanish) of this measure has been established with Latinx populations (Knight et al. 1994). Mothers rated items on a 5-point scale (1 = *Almost Never* to 5 = *Almost Always*) at separate points in the interview for 5th graders and younger siblings (counterbalanced), which were averaged for the scale score. Cronbach’s alphas were 0.81 and 0.78 for mothers’ warmth with 5th graders and younger siblings, respectively.

Mothers also rated the frequency of conflict in nine domains with each child (i.e., 5th graders, younger siblings) on a 6-point scale ranging from 1 = *Not at all* to 6 = *Several times a day*. Items were originally based on a measure by Smetana (1988) and adapted for Latinx families (Updegraff et al. 2009). Domains of conflict included homework/schoolwork, respect for parents, getting along with sisters/brothers, doing chores, electronics, bedtime and curfew, friends, and the importance of family. Items were rated at separate points in the interview for 5th graders and younger siblings (counterbalanced) and averaged to create a mother–child conflict score with each child. Cronbach’s alphas were 0.86 and 0.85 for mothers’ ratings with 5th graders and younger siblings, respectively.

#### 2.3.6. Children’s Educational Adjustment

Teachers rated children’s academic and social competencies (Conduct Problems Prevention Research Group 1999) and aggressive/oppositional behaviors (Werthamer-Larsson et al. 1991) in the school setting. The academic competence subscale included five items assessing behaviors such as reading grade-level materials, solving grade-level math problems, and turning in homework. The socioemotional competence subscale included 12 items reflecting prosocial behaviors (e.g., “This child shows empathy and compassion for others’ feelings”) and emotion regulation (“This child recognizes and labels his/her feelings and those of others appropriately”). The aggressive/oppositional behaviors subscale (7 items) included behaviors such as breaking things on purpose, yelling at others, and knowingly breaking the rules. Teachers were instructed to rate the target child relative to other students in the same grade level on all three subscales ranging from 1 (*Almost Never*) to 5 (*Very Often*). Items were averaged to create mean scores for academic competence (αs = 0.90 for 5th graders and 0.92 for younger siblings), socioemotional competence (αs = 0.76 for 5th graders and 0.72 for younger siblings), and aggressive/oppositional behavior subscales (αs = 0.93 for 5th graders and 0.95 for younger siblings).

### 2.4. Covariates

Mothers reported on their nativity status (0 = U.S.-born; 1 = born outside the U.S.) and children’s gender (0 = male; 1 = female). Children’s age was calculated based on their date of birth and the home visit date. Birth order was coded as 0 = 5th grader (older sibling) and 1 = younger sibling.

## 3. Results

### 3.1. Preliminary Analyses

Means, standard deviations, and bivariate correlations are shown in Table 1. Examination of mean scores indicated that, on average, Latinx mothers reported moderately high levels of familism values and family cohesion, falling above the midpoint on both scales; their experiences of economic hardship were slightly below the midpoint, and experiences of discrimination were relatively low (i.e., 1.5 on a 5-point scale). In terms of the parent–child relationship, warmth was well above the midpoint and conflict was slightly below the midpoint. Teachers’ ratings of children’s academic competencies revealed that children were between “sometimes” and “often” able to perform at grade level, achieve academic goals, and turn in homework, and that they demonstrated moderately high levels of socioemotional competencies and relatively low levels of aggressive/oppositional behaviors. Bivariate correlations were generally in expected directions and small to medium in size based on Cohen (1992), with values of 0.20, 0.50 and 0.80 indicating small, medium, and large correlations, respectively. Among mothers’ cultural assets and challenges, familism values and cohesion were positively correlated as were discrimination and economic hardship, with both being small in magnitude. Links between mothers’ assets/stressors and mother–child warmth and conflict were in expected directions, with cultural assets being associated with more warmth and stressors being associated with more conflict. Among the three indicators of children’s educational adjustment, correlations were medium in size, ranging from 0.43 to 0.65. Paired *t*-tests revealed a significant birth order difference in mother–child warmth, *t* (173) = 2.34, *p* = 0.02, with mothers reporting more warmth with younger compared to older siblings (see Table 1 for means), but there were no mean differences between older and younger siblings for mother–child conflict or educational adjustment outcomes.

### 3.2. Associations among Mothers’ Cultural Assets and Challenges, Parent–Child Relationships, and Children’s Educational Adjustment

**Figure 1.**
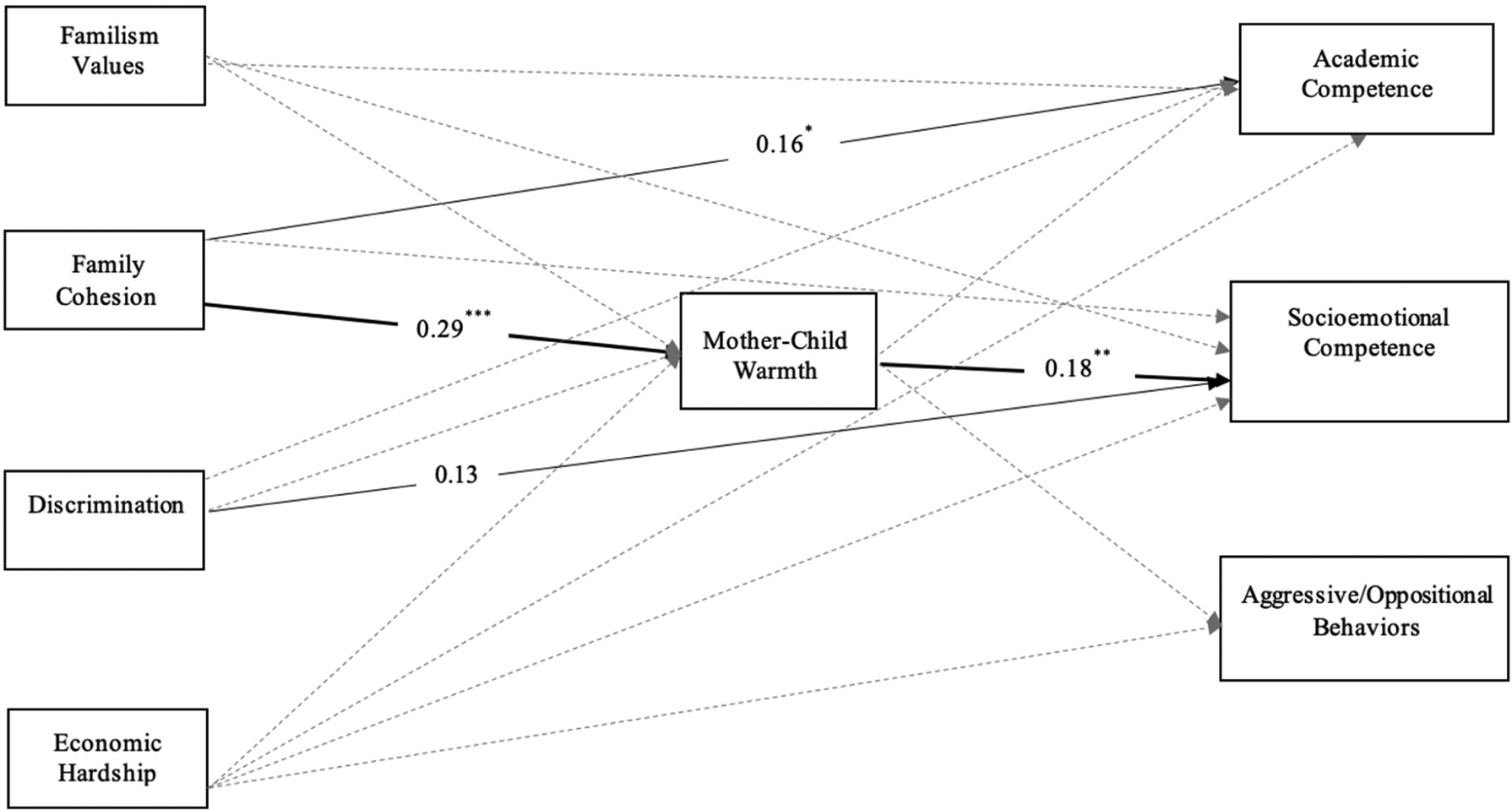
Mothers’ Cultural Assets and Challenges and Children’s Educational Adjustment via Mother–Child Warmth. Note. Standardized parameters are reported. Covariances, residual correlations, and covariates are not shown in the figure for parsimony. Solid lines represent significant direct effects. Bolded lines represent significant indirect paths. Dashed lines represent nonsignificant paths. * *p* < 0.05, ** *p* < 0.01, *** *p* < 0.001.

**Figure 2.**
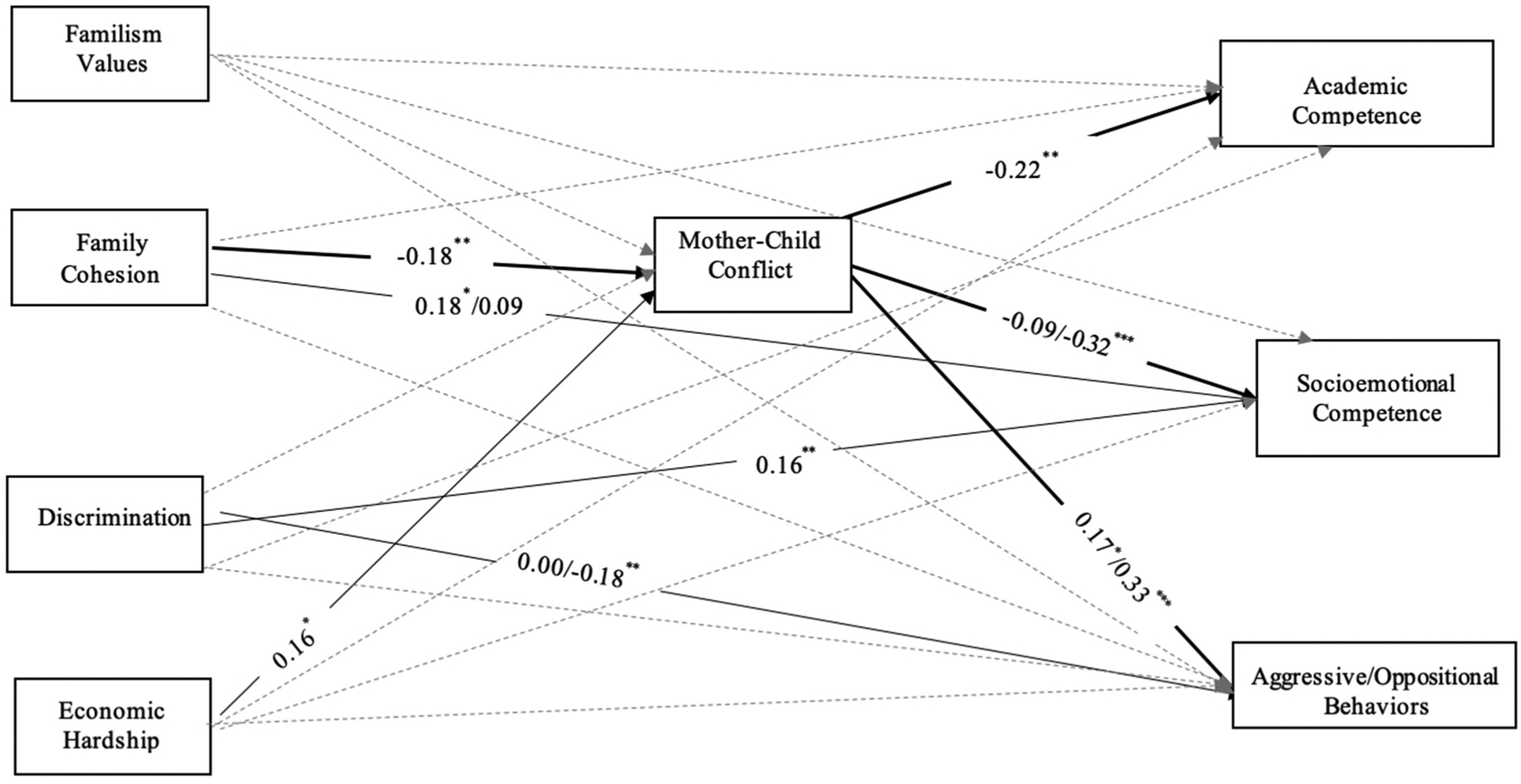
Mothers’ Cultural Assets and Challenges and Children’s Educational Adjustment via Mother–Child Conflict. Note. Standardized parameters are reported. For paths that differed by sibling birth order, parameters are reported for each group separated by the slash symbol (older sibling/younger sibling). Covariances, residual correlations, and covariates are not shown in the figure for parsimony. Solid lines represent significant direct effects. Bolded lines represent significant indirect paths. Dashed lines represent nonsignificant paths. * *p* < 0.05, ** *p* < 0.01, *** *p* < 0.001.

Path analytic models were conducted in Mplus (version 8.6; Muthén and Muthén 1998–2018) to test direct effects of Latinx mothers’ familism values, family cohesion, economic hardship, and discrimination on children’s academic competence, socioemotional competence, and aggressive/oppositional behaviors, as well as the indirect effects via mother–child warmth and conflict. Covariates included mother nativity, birth order, child gender, and child age. Two parallel models were tested, one for mothers’ reports of warmth and a second for their reports of conflict. Missing data ranged from 0% to 9% across study variables and were estimated via full information maximum likelihood. To adjust for the nonindependence of observations due to the nestedness of siblings within families, we used the TYPE = COMPLEX command (Muthén and Muthén 1998–2018). Statistical significance of indirect effects was estimated using 1000 bootstrap resamples and examination of confidence intervals (MacKinnon et al. 2004). Moderation by child gender and birth order were examined using multiple group analysis. Specifically, child gender was removed as a covariate and the model with freely estimated paths across boys and girls was compared against a fully constrained model with all regression paths set to be equal across groups. When constraining all structural paths to be equal across groups did not yield a significant decrease in model fit based on the Satorra–Bentler adjusted chi-squared difference test, the constrained model was retained for parsimony (Satorra and Bentler 2001). When constraining all paths produced a significant decrease in the model fit, each parameter estimate was compared using the MODEL CONSTRAINT command to identify paths that significantly differed across groups to achieve a final partially constrained model. The process was repeated to test sibling birth order moderation. Unstandardized coefficients are reported in the text and standardized coefficients are shown in Table 2 and Figures 1 and 2.

For the model including mother–child *warmth*, comparisons of the fully unconstrained and constrained models by child gender and birth order revealed no changes in model fit, Δχ^2^ (19) = 9.82, *p* = 0.957 for child gender, and Δχ^2^ (19) = 26.98, *p* = 0.105 for sibling order, suggesting that the structural paths did not differ for girls versus boys and older versus younger siblings. As shown in Figure 1, family cohesion was positively related to academic competence (*b* = 0.30, *p* = 0.012), mothers’ experiences of discrimination were positively associated with child socioemotional competence (*b* = 0.09, *p* = 0.014), and familism and perceived economic hardship were not significantly associated with children’s adjustment. There was one significant indirect effect in this model: family cohesion was associated with socioemotional competence via mother–child warmth (*ab* = 0.052, 95% CI = [0.021, 0.096]). Significant covariates included child gender predicting academic competence (*b* = 0.30, *p* = 0.008), socioemotional competence (*b* = 0.24, *p* < 0.001), and aggressive/oppositional behaviors (*b* = −0.43, *p* < 0.001), such that girls reported higher academic and socioemotional competencies than boys, and boys reported higher aggressive/oppositional behaviors than girls. Mother nativity predicted academic competence (*b* = 0.48, *p* = 0.002), socioemotional competence (*b* = 0.17, *p* = 0.012), and aggression (*b* = −0.20, *p* = 0.029), such that teachers reported higher academic and socioemotional competencies and lower aggressive/oppositional behaviors for children of mothers born outside the U.S. Child age was not a significant covariate of any of the three adjustment outcomes.

For the second model including mother–child *conflict*, there were no differences between the fully unconstrained and constrained models by child gender, Δχ^2^ (19) = 23.72, *p* = 0.207, but significant differences were found for sibling birth order, Δχ^2^ (19) = 38.87, *p* = 0.005. Testing specific paths revealed the following birth order differences: family cohesion to child socioemotional competence, mothers’ discrimination to aggressive/oppositional behavior, and mother–child conflict to socioemotional competence and aggressive/ oppositional behavior. Thus, these paths and their corresponding indirect effects were allowed to vary for older versus younger siblings, while all other structural paths were constrained to be equal (see Figure 2). Family cohesion was positively associated with children’s socioemotional competence for older siblings (*b* = 0.17, *p* = 0.027), but no significant association emerged for younger siblings (*b* = −0.08, *p* = 0.166). Mothers’ reports of discrimination were associated with children’s socioemotional competence for both older and younger siblings (*b* = 0.12, *p* < 0.001). Mothers’ discrimination also was negatively associated with aggressive/oppositional behaviors for younger siblings (*b* = −0.21, *p* = 0.011), but no significant association emerged for older siblings (*b* = −0.003, *p* = 0.972). Mothers’ familism and economic hardship did not significantly predict children’s educational adjustment, but economic hardship was positively related to mother–child conflict (*b* = 0.26, *p* = 0.024).

Turning to indirect effects, mother–child conflict had significant indirect effects on the relations between family cohesion and children’s academic competence (*ab* = 0.075, 95% CI = [0.019, 0.158]) for both older and younger siblings. In addition, the indirect effect of mother–child conflict was significant for family cohesion and aggressive/oppositional behaviors (*ab* = −0.09, 95% CI = [−0.184, −0.027]) and family cohesion and socioemotional competence (*ab* = 0.049, 95% CI = [0.014, 0.094]) for younger siblings, but not for older siblings (*ab* = −0.038, *p* = 0.119 for aggressive/oppositional behaviors and *ab* = 0.016, *p* = 0.233 for socioemotional adjustment). In terms of covariates, child gender was a significant predictor of socioemotional competence (*b* = 0.22, *p* = 0.002) and aggressive/oppositional behaviors (*b* = −0.32, *p* < 0.001); mother nativity predicted academic competence (*b* = 0.47, *p* = 0.01), such that children with mothers born outside of the U.S. had higher academic competence relative to those with U.S.-born mothers. Child age was not a significant covariate.

## 4. Discussion

The 21st century has brought unprecedented change in the ethnic–racial composition of the U.S. (Krogstad 2019), highlighting the importance of examining the opportunities and challenges parents face, particularly parents from ethnic–racial minoritized groups who have been historically underrepresented in research and prevention science. Our focus was on one such group, Latinx families, whose children comprise more than 25% of the K-12 school population (Child Trends 2018). This study contributes new knowledge about the associations among mothers’ culturally based assets and challenges, universally salient features of the mother–child relationship (i.e., warmth, conflict), and children’s educational adjustment based on a sample of predominantly immigrant Latinx families. Our focus on middle childhood addresses a significant gap, as most research on Latinx families has examined parenting dynamics in *adolescence*. Indeed, middle childhood is a time when prevention efforts, particularly supports for parents (e.g., Parra Cardona et al. 2012), may be critical in shaping children’s educational adjustment and, subsequently, their long-term educational and economic opportunities (National Center for Education Statistics 2021).

### 4.1. Culturally Informed Assets, Mother–Child Relationship Quality, and Children’s Educational Adjustment

Guided by culturally and ecologically based models (Calzada et al. 2012; García Coll et al. 1996; Stein et al. 2014), we examined mothers’ familism values and family cohesion as potential resources that may be directly associated with children’s academic, socioemotional, and behavioral functioning and indirectly linked via mother–child relationship quality. Our findings revealed the expected positive bivariate associations between mothers’ familism values and mother–child warmth (Cahill et al.; Stein et al. 2014), but there were no significant direct associations from mothers’ familism values to children’s educational adjustment. These nonsignificant associations stand in contrast to research with primarily *adolescent* and *young adult* samples, revealing links between familism values and more positive educational outcomes and fewer externalizing symptoms (Cahill et al.). There also was no evidence that mothers’ familism values were indirectly related to children’s educational adjustment via mother–child warmth and conflict. When accounting for multiple dimensions of mothers’ cultural assets and challenges within a single model, *family cohesion* was directly and indirectly associated with children’s educational adjustment. Specifically, family cohesion was related to higher levels of mother–child warmth, which was associated with more positive socioemotional functioning. That this finding was consistent across sibling birth order and child gender suggests the general importance of family cohesion and mother–child warmth for children’s socioemotional competencies (i.e., prosocial interactions, emotional regulation strategies) in elementary school, and is consistent with research on recent Latinx immigrant families with slightly older children (i.e., 9 to 12 years of age; (Leidy et al. 2010)). In this sample of predominantly immigrant Latinx mothers who endorsed strong family-oriented values (i.e., almost 4.5 on a 5-point scale), it is possible that family interactions that are consistent with these values, including shared activities, interests, and connectedness among family members, along with warm and supportive relations between mothers and their children, are particularly salient for children’s socioemotional functioning (Leidy et al. 2010). Our findings suggest the importance of examining both attitudinal *and* behavioral components of familism across different developmental periods to gain a more complete picture of their implications for family functioning and youth adjustment (Cahill et al.; Stein et al. 2014).

These findings also complement research on Latinx parenting styles in middle childhood (Domenech Rodriguez et al. 2009), which has identified different constellations of parenting behaviors (warmth, demandingness, and autonomy granting). Further, we extend research on the benefits of family cohesion and parental support for Latinx adolescents’ adjustment (Bámaca-Colbert et al. 2018) to middle childhood (Leidy et al. 2010) and to children’s positive socioemotional functioning in the school setting. Although our findings cannot be generalized to other sociocultural contexts, it is possible that family cohesion and warm and supportive mother–child relationships in middle childhood are resources that benefit all children, regardless of their culturally and contextually based resources and challenges, as they navigate their early years of formal schooling and learn to interact with teachers and classmates.

Turning to the models including mother–child conflict as a potential indirect mechanism, our findings extend research that has predominantly focused on parent–youth conflict in Latinx families during *adolescence* (e.g., Li and Warner 2015; Padilla et al. 2016; Pasch et al. 2006). For both older and younger siblings and female and male children, higher levels of family cohesion were associated with lower levels of mother–child conflict and, in turn, teachers’ reports of children’s more positive academic functioning, specifically their completion of homework and ability to achieve their academic goals and master grade-level reading and math skills. A home environment characterized by family connectedness and low levels of mother–child conflict may increase the likelihood that Latinx children can focus on school-related tasks and be successful in their academic work. These more harmonious family environments, which align with the cultural values of Latinx families (Campos et al. 2008; Leidy et al. 2010), may provide the foundational skills and motivation for children to be successful at school, highlighting potential sources of strength and resilience for Latinx youth (Cahill et al.; Leidy et al. 2010; Stein et al. 2014). More generally, these findings underscore the importance of the family context in children’s successful academic functioning in *middle childhood*, a critical time when they are adapting to a complex educational environment (Collins and Madsen 2019; Votruba-Drzal 2006) that is unfamiliar to the majority of their parents, who have limited experience with U.S. K-12 schools (Suarez-Orozco et al. 2010).

It is notable that the pattern of findings linking family cohesion to mother–child conflict to children’s adjustment was significant for *all* three domains of educational adjustment for younger siblings. That is, for younger siblings the indirect effects emerged for socioemotional competence and aggressive/oppositional behaviors in addition to academic competence, suggesting the particular importance of these familial processes for younger children during their early to middle elementary school years (i.e., 1st to 4th grade). A strength of our study was the focus on two children in each family and the consideration of within-family variation. Although family cohesion was related to less mother–child conflict for both siblings, and mothers reported similar levels of conflict with older and younger siblings, the links from mother–child conflict to children’s socioemotional functioning and aggressive/oppositional behaviors were specific to younger siblings. It is possible that mother–child conflict has different implications for the adjustment of older siblings, who are on the verge of adolescence, relative to their younger siblings who are in middle childhood. One possibility is that, for younger siblings, who are in middle childhood and have relatively less autonomy and spend more time with parents than their older siblings (Shanahan et al. 2007a), mother–child conflicts may be more salient for their adjustment. It also may be that mother–child conflicts in middle childhood are more likely to reflect interpersonal difficulties that also manifest in children’s interactions at school, including more limited emotion regulation skills and more frequent aggressive/oppositional behaviors. These findings are among the first to examine within-family variation in these processes in middle childhood in Latinx families, and underscore the potential importance of mother–child conflict in middle childhood in this cultural context, particularly for younger children in our sample. Furthermore, our findings suggest the possibility that prevention programs that include strategies for addressing mother–child conflict in middle childhood may be beneficial for Latinx children’s functioning in multiple domains at school.

### 4.2. Culturally Informed Challenges, Mother–Child Relationship Quality, and Children’s Educational Adjustment

In addition to considering the strengths that Latinx mothers bring to their parenting, we also considered two particular challenges that are disproportionately experienced by Latinx families: economic hardship (Shrider et al. 2021) and discrimination (Ayón and Garcia 2019). Our expectations regarding the associations among stressors, parent–youth relationship quality, and children’s educational adjustment were informed by the family stress model (Conger et al. 1994) and its extension to culturally based stressors (Conger et al. 2012; Lorenzo-Blanco et al. 2012). Our measure of economic hardship focused on mothers’ perceptions of how difficult it was to meet their financial demands, such as paying bills on time, providing essential items such as food and clothing, and covering medical expenses. As our sample was recruited from economically disadvantaged schools, the distribution of household income was limited in range and less likely to provide an indication of whether and how economic circumstances are associated with stress and hardship for families. We found that mothers’ perceptions of family economic hardship were positively related to mother–child conflict at the bivariate level and in our multivariate model for both older and younger siblings. However, contrary to the family stress process model and empirical support with Latinx families in early childhood (Derlan et al. 2019) and early adolescence (Delgado et al. 2013; White et al. 2015), we found limited evidence linking mothers’ perceptions of economic hardship to children’s adjustment via mother–child relationship quality in middle childhood.

Several characteristics of this study may underlie the lack of support for the family stress model. First, mothers’ ratings of economic hardship were low to moderate, and all children attended Title I elementary schools where additional financial supports may be widely in place. Most families (almost 98%) also accessed various sources of public assistance (e.g., free/reduced price meals, nutritional assistance, and healthcare). Thus, in the context of these safety nets and mothers’ modest reports of hardship (despite fairly low median income in households supporting an average of six individuals), economic hardship may not be a particularly salient challenge for children’s educational adjustment. Developmental considerations also may play a role here. As children in middle childhood spend more time in the home setting relative to youth in adolescence (Shanahan et al. 2007b), and in this sample they also spent their school days with children in similar economic circumstances, this younger age group may be less aware of or affected by the economic hardship experienced in their homes. Notably, much of the research on the family stress model with Latinxs focuses on families with adolescents (e.g., Delgado et al. 2013; White et al. 2015), who may be more cognizant of their families’ economic hardships to the extent that they have more opportunities to compare and contrast their situations to those of their peers. Along these lines, Delgado et al. (2013) showed that *adolescents’ perceptions* of their families’ economic hardship mediated parents’ reports of hardship to family functioning and adolescents’ adjustment. Thus, one direction of future research may be to assess the degree to which children perceive economic constraints or stressors within the family context and the role of these perceptions in their educational adjustment.

Turning to our second indicator of culturally based stress, mothers’ experiences of discrimination, our findings revealed inconsistencies across the bivariate and multivariate analyses. At the bivariate level, mothers’ reports of discrimination were associated with greater economic hardship and more mother–child conflict for older and younger siblings, consistent with the premises of the family stress model (Conger et al. 2012; Lorenzo-Blanco et al. 2012). In the multivariate model, discrimination was associated with more positive socioemotional competencies for both older and younger siblings and less aggressive/oppositional behavior for younger siblings. Although mothers who experience more frequent discrimination may engage in socialization strategies (e.g., preparation for bias, cultural socialization; Hagelskamp and Hughes 2014) that have been demonstrated to promote positive adjustment among children (Umaña-Taylor and Hill 2020), replication is essential, as it is also likely that these findings are spurious. Further, we know little about the potential underlying *mechanisms* linking Latinx mothers’ experiences of discrimination to their children’s adjustment in middle childhood, highlighting an important direction of future work.

## 5. Limitations and Future Directions

This study has several limitations that guide future research. Foremost, our study drew on cross-sectional data and future longitudinal work is needed to test for potential direct and indirect mechanisms using multiple time points and accounting for prior levels of children’s adjustment. Although our findings highlight important contextual and parenting characteristics that are linked to children’s educational adjustment, the direction of the effects cannot be determined. Second, because our study required the participation of a primary caregiver, and the majority were biological mothers, future work should consider how multiple caregivers’ cultural assets and challenges are linked to children’s adjustment. This should include father figures and other primary caregivers in the lives of Latinx children (e.g., grandparents, aunts and uncles, older siblings; Updegraff and Perez-Brena). Third, we controlled for mothers’ nativity as a proxy for their exposure to anti-immigrant experiences. The associations between mothers’ nativity and children’s educational adjustment suggested, however, that mothers’ immigrant status (i.e., being born outside of the U.S.) was related to more positive child adaptation in school, aligning with findings regarding the immigrant paradox (Gonzales et al. 2009). Thus, it will be important to directly measure the extent to which parents have been the target of anti-immigrant actions and policies in future studies, given the salience and frequency of such experiences for Latinx families in the 21st century (Roche et al. 2018; Torres et al. 2018). Fourth, we drew on mothers’ reports of parent–child relationship qualities, and the next steps should examine how children perceive these relationships as well as use observational ratings of parent–youth relationships (e.g., Domenech Rodriguez et al. 2009). Finally, our analytic sample of 348 children from 173 families was large for a primary data collection effort, but nevertheless underpowered for a complex model testing moderation by birth order and gender. On one hand, this suggests that the associations that emerged are likely quite robust; on the other hand, more research with larger samples is needed, as some of our null findings could be due to limited power to detect significant associations.

These limitations withstanding, this study has a number of strengths and makes several important contributions to the field. For instance, a feature of our study that strengthens its external validity is its multiple-informant design, in which we gathered data from teachers to assess children’s educational adjustment, rather than relying on mothers’ reports; this enables us to rule out common source variance as an explanation for our results. Our study also provided important evidence regarding the promotive function of family cohesion for the educational adjustment of Latinx children. Among the culturally based assets and challenges examined, *family cohesion* was a particularly important familial asset in middle childhood that was associated with more positive mother–child relationship dynamics and, in turn, with more optimal adjustment at school. The pattern of findings was particularly strong for younger siblings, who are early in their years of formal schooling—a time when establishing a foundation for future educational success has far reaching benefits (Magnuson et al. 2016; Rabiner et al. 2016).

More generally, our study underscores the interconnections between family and school experiences and suggests that prevention and intervention programs that focus on these intersecting contexts have the potential to benefit Latinx youth. As one example, Gonzales et al. (2012) demonstrated the efficacy of a school-based, parent–adolescent-focused prevention program (i.e., *Bridges to High School Program/Projecto Puentes a la Secundaria*) designed to increase school engagement and reduce risk behaviors by strengthening parenting, adolescent coping, school engagement, and family cohesion among Mexican-origin families in urban school settings. Investing in programs that build family–school connections and enhance family-based supports *prior* to adolescence (and the onset of significant behavioral difficulties for some youth) may be an important next step in prevention research to capitalize on a developmental period when children spend the majority of their time in family and school settings.

## 6. Conclusions

In the 21st century, Latinx families account for more than half of the growth in the U.S. population and represent more than one-fourth of the school-age population. One key contribution of our research, and a critical approach to studying Latinx families in the 21st century, is a balanced perspective that recognizes the strengths *and* challenges of Latinx families in the U.S. today. In considering multiple assets and challenges, our work underscored *family cohesion* as a salient cultural factor in middle childhood that is linked to more positive family dynamics and, in turn, more positive adaptation in the school setting. Prevention programs that build on these familial strengths have the potential to support children’s successful transition to and adjustment in their early years of formal schooling. A particularly important contribution was our focus on *middle childhood*, a period of parenting that has been relatively neglected in research on Latinx parenting/parent–child relationships. Middle childhood is a potentially ideal time to intervene with efficacious prevention programming, as it could help set children on a more positive trajectory as they enter adolescence and aid in addressing the considerable disparities in high school and postsecondary education for ethnic–racial minoritized and low-income youth. The potentially different pattern of findings that emerged in this middle childhood sample, compared to a body of research on parenting among Latinx adolescents, underscores the importance of ensuring that research on Latinx families spans children’s development from early childhood to young adulthood to better understand the unique resources and challenges that characterize each developmental period. Continuing to build a foundation of knowledge on Latinx families will be essential to informing culturally responsive prevention and intervention programming in the 21st century.

## Figures and Tables

**Table 1.** Bivariate Correlations, Means, and Standard Deviations for Study Variables.

	1	2	3	4	5	6	7	8	9	10	11	12
1. Fam	-	0.28 ***	0.12	0.09	0.21 **	−0.03	−0.04	−0.02	0.04	0.02	0.12	0.13
2. Coh	0.28 ***	-	0.04	−0.18 *	0.35 ***	−0.24 *	0.06	−0.07	−0.04	−0.02	−0.13	0.09
3. Disc	0.12	0.04	-	0.22 **	0.05	0.16 *	0.05	0.13	0.1	0.09	−0.04	0.13
4. Econ	0.09	−0.18 *	0.22 **	-	−0.14	0.19 *	0.03	0.02	0.1	0	0.06	−0.13
5. Warm	0.19 *	0.36 ***	−0.06	−0.16 *	-	−0.34 ***	0.05	0.19 *	−0.18 *	0.05	−0.06	0.09
6. Conf	0.03	−0.19 *	0.21 **	0.25 **	−0.31 ***	-	−0.29 ***	−0.32 ***	0.35 ***	−0.16 *	0.08	−0.21 **
7. Acad	0.03	0.23 **	0.04	−0.13	0.04	−0.24 **	-	0.59 ***	−0.49 ***	0.20 *	−0.07	0.17 *
8. Socio	0.01	0.21 **	0.01	−0.06	0.19 *	−0.11	0.45 ***	-	−0.65 ***	0.29 ***	0.08	0.16 *
9. Agg	−0.03	−0.09	0.11	0.06	−0.08	0.20 **	−0.43 ***	−0.47 ***	-	−0.33 ***	−0.03	−0.1
10. Gen	0.06	0.07	0.09	0.01	0	−0.03	0.08	0.20 **	−0.24 **	-	0.11	0.02
11. Age	0.04	−0.1	0.12	−0.02	0.03	0.02	−0.06	0.08	0.1	−0.11	-	−0.04
12. Nat	0.13	0.09	0.13	−0.13	0.11	−0.17 *	0.23 **	0.12	−0.15	−0.07	0.07	-
OS *M* (*SD*)	4.41 (0.43)	3.97 (0.54)	1.50 (0.45)	1.72 (0.61)	4.44 (0.49)	2.40 (0.99)	3.48 (0.98)	3.39 (0.50)	1.44 (0.67)	0.47 (0.50)	10.63 (0.41)	0.73 (0.44)
YS *M* (*SD*)	4.41 (−0.43)	3.97 (0.54)	1.50 (0.45)	1.72 (0.61)	4.52 (0.44)	2.35 (1.00)	3.46 (1.06)	3.44 (0.46)	1.47 (0.81)	0.53 (0.50)	8.25 (1.06)	0.73 (0.44)

Note. Correlations are above the diagonal for younger siblings and below for older siblings. Fam = Familism, Coh = Cohesion, Disc = Discrimination, Econ = Economic hardship, Warm = Mother-child warmth, Conf = Mother-child conflict, Acad = Academic competence, Socio = Socioemotional competence, Agg = Aggressive/Oppositional Behaviors, Gen = Child gender, Age = Child age, Nat = Mother nativity; Mother nativity is coded as 0 = *U.S.-born* and 1 = *born outside the U.S*.; children’s gender coded as 0 = *male* and 1 = *female*. *M* = Mean, *SD* = Standard Deviation.

* *p* < 0.05,

** *p <* 0.01,

*** *p <* 0.001.

**Table 2.** Results for the associations between mothers’ cultural assets and challenges and children’s educational adjustment via mother–child warmth and conflict.

	Mother-Child Warmth Model		Mother-Child Conflict Model	
	β	SE	β	SE
**Predictors of Mother-Child Warmth/Conflict**				
Familism values	0.121	0.063	0.017	0.054
Family cohesion	0.294 ***	0.059	−0.184 **	0.067
Discrimination	−0.030	0.045	−0.141 *	0.071
Economic hardship	−0.119	0.061	0.157 *	0.068
**Predictors of Academic Competence**				
Mother-child warmth/conflict	−0.019	0.073	−0.223 **	0.065
Familism values	−0.091	0.066	−0.080	0.067
Family cohesion	0.159 *	0.062	0.116	0.063
Discrimination	−0.076	0.062	−0.104	0.064
Economic hardship	−0.002	0.065	0.022	0.065
Mother nativity	0.207 **	0.066	0.211 **	0.081
Child gender	0.149 **	0.055	0.090	0.075
Child age	−0.013	0.051	−0.038	0.072
**Predictors of Socioemotional Competence**				
Mother-Child warmth/conflict	0.177 **	0.061	−0.091/−0.318 ***	0.066/064
Familism values	−0.104	0.056	−0.07	0.086
Family cohesion	0.041	0.060	0.184 */−0.092	0.054/065
Discrimination	−0.128 *	0.050	−0.162 **	0.048
Economic hardship	0.010	0.053	0.011	0.047
Mother nativity	0.154 *	0.062	0.124	0.088
**Predictors of Socioemotional Competence**				
Child gender	0.25 ***	0.047	0.222 **	0.064
Child age	0.021	0.053	0.139	0.079
**Predictors of Aggressive/Oppositional Behaviors**				
Mother-Child warmth/conflict	−0.104	0.070	0.166 */0.325 ***	0.078/0.077
Familism values	0.074	0.061	0.044	0.064
Family cohesion	−0.020	0.058	0.008	0.059
Discrimination	0.072	0.057	0.003/−0.181 **	0.089/0.068
Economic hardship	0.059	0.062	0.048	0.065
Mother nativity	−0.121	0.059	−0.132	0.078
Child gender	−0.29 ***	0.044	−0.236 ***	0.060
Child age	−0.033	0.051	0.075	0.070

Note. Estimates that differed by sibling birth order are reported in the order of older/younger sibling.

* *p* < 0.05,

** *p* < 0.01,

*** *p* < 0.001.

## Data Availability

Contact the corresponding author regarding requests for data.
